# Supplementary material for: Thermodynamically consistent determination of free energies and rates in kinetic cycle models
Source: bioRxiv. 2023 Apr 9:2023.04.08.536126. Preprint. [Version 2] doi: 10.1101/2023.04.08.536126 (PMC10104237; doi:10.1101/2023.04.08.536126)
Supplement: Supplementary file 1 [file media-1.pdf]

Article

Supplementary Information: Thermodynamically consistent determination of free energies and rates in kinetic cycle models

Ian Kenney[1] and Oliver Beckstein[1,2]

[1]Arizona State University, Department of Physics, Tempe AZ, USA
[2]Arizona State University, Center for Biological Physics. Tempe AZ, USA

1 MICROSCOPIC PROTONATION STATES OF DTPA FROM NMR DATA

1.1 NMR data

NMR data for the chemical shifts of the central and terminal nitrogens of diethylenetriaminepentaacetic acid (DTPA) were extracted from Figure 4 of Submeier and Reilley (1) using the WebPlotDigitizer tool(2). The raw data are reported in Table S1 and available as CSV files from the data repository (see main paper). Each data set was extracted independently. Note that the difference in number of data points was also present in the original data.

pH (central)	ppm (central)	pH (terminal)	ppm (terminal)
3.1005291729704183	3.6928879974260154	3.141767697857979	3.9610068662417772
3.500318325266809	3.681291373727555	3.523593721119683	3.961438145635521
4.0082148455951305	3.615599991785155	3.9778746825304463	3.9468910232274763
4.588237710248704	3.516857547731676	4.304880302853974	3.9322001410215166
4.877619337746529	3.432847060112133	4.577503645337732	3.9264839776282376
5.16787721544117	3.3970303333173604	4.886381839714669	3.914784668359838
5.657974903646707	3.352403184621809	5.177242139419619	3.912101152132095
6.312259972480266	3.3380819704676306	5.68639826940586	3.9156883013753023
6.784942188024123	3.335603825379766	6.304592783258144	3.9163865632508887
7.075857253366374	3.335932419203571	6.795183362199387	3.898868405019271
7.494321487982364	3.351465323083032	7.086098427541637	3.899196998843076
7.804021166918816	3.384947664587855	7.503686411960814	3.866536141897767
8.022481294112008	3.4002546602134496	7.812181246876646	3.83375206226853
8.259123612889091	3.415582192953032	8.047892549819615	3.7978737241317937
8.587279311595939	3.4641456218295836	8.265640723727895	3.7740232890872627
8.89703375616969	3.5006400733859544	8.591277203118903	3.6840296555925987
9.479192480677998	3.5193699213428538	8.897964772003805	3.551845944262273
10.005216426952908	3.4506869664628934	9.531877023761439	3.41701979093218
10.512236697084413	3.3368018236957226	10.003299629647376	3.3452631146587075
11.601306160449624	3.235622308782355	10.493452083490212	3.303648076014705
12.510196677094957	3.224600724275554	11.601689519910732	3.256707079143192
		12.492616907521374	3.2577133977285957

Table S1: Raw hydrogen chemical shift data of *central* and *terminal* nitrogen atoms in DTPA from 1D NMR, extracted from Figure 4 in Submeier and Reilley (1).

Each data set in in Table S1 was normalized by subtracting the minimum chemical shift from all entries and dividing by the resulting maximum.

1.2 Inverse *Multibind* for NMR data

In order to constrain the *Multibind* model for DTPA we used the normalized 1D NMR shift data (based on data in Table S1) and interpreted the titration curves as site-resolved mean proton occupancy. The goodness-of-fit of the model to the experimental data was measured by the RMSD between the computed and the experimental titration curve. The RMSDs for different training data sets (only central nitrogen, only terminal nitrogens, or all central and terminal nitrogen) to the other data sets are shown in Table S2.

Training data	$RMSD_{terminal}$	$RMSD_{central}$	$RMSD_{all}$
terminal	0.012	**0.094**	0.066
central	**0.067**	0.017	0.049
all	0.030	0.027	0.029

Table S2: Root-mean-square deviation (RMSD) of the calculated mean proton occupancy curves to experimental NMR data. The *Training data* indicates which data set was used for fitting the model. The RMSD to either the *terminal* or the *central* nitrogen data or the combined (*all*) data is computed. RMSD values in **bold** are considered validation as the experimental comparison data set was not used for training.

The microscopic pK_a values of the inverse *Multibind* model were reported in the main paper when all experimental data were used for training. Table S3 shows the pK_a values also for the reduced training data sets (*central* and *terminal*).

State 1	State 2	Central[a]	Terminal[b]	All[c]
000	001	9.3	9.5	9.0
000	010	10.1	10.2	10.1
000	100	9.3	9.5	9.0
001	011	8.4	8.4	9.0
001	101	9.3	9.4	9.8
010	011	7.6	7.7	7.9
010	110	7.6	7.7	7.9
100	101	9.3	9.4	9.8
100	110	8.4	8.4	9.0
011	111	5.6	5.5	5.5
101	111	4.7	4.4	4.7
110	111	5.6	5.5	5.5

[a] Fit was performed only on the titration curve of the central nitrogen atom.
[b] Fit was performed only on the titration curve for the terminal nitrogen atoms.
[c] Fit was performed on the titration curves of both the central and terminal nitrogen atoms.

Table S3: Microscopic pK_a values for DTPA from the inverse *Multibind* approach, split by experimental training data. States are labeled as described in the main paper.

2 ANTIPORTER MODEL

The process for developing the model of a sodium/proton antiporter is shown in Fig. S1. We started by defining states, based on the inward facing (IF) and outward facing (OF) conformations and binding of individual ions (3).

2.1 Model parameters

To define the equilibrium system we chose binding constants for sodium ions, measured by K_D, and pK_as for protons (estimated parameters in Table S4). The free energy differences between the IF and OF conformations were calculated from the rates in

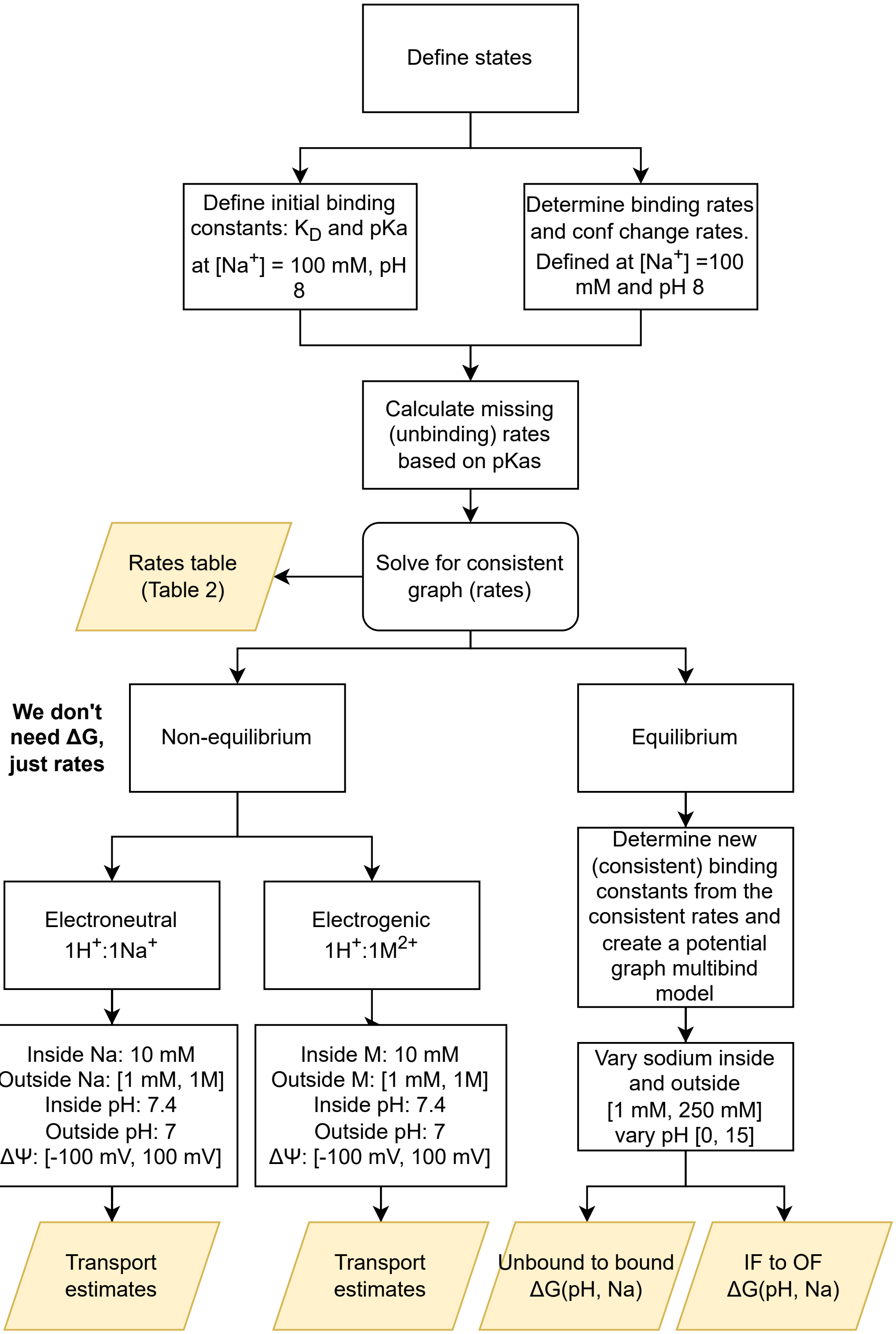

Figure S1: Flowchart following the development of both equilibrium and non-equilibrium models of a toy sodium/proton antiporter.

conformation	Na^+	H^+
	estimated parameters	
	$\overline{K_D}$	$\overline{pK_a}$
Inward	22 mM	6.4
Outward	53 mM	6.5
	thermodynamically consistent parameters	
	K_D	pK_a
Inward	23.98 mM	6.44
Outward	52.21 mM	6.51

Table S4: Equilibrium binding constants (K_D for sodium ions and pK_a for protons) in the sodium/proton antiporter model.

Table 2, which had been chosen so that the free energy differences were $< 1kT$. Because we are also interested in modeling transport out of equilibrium we required forward and backward rates for ion binding processes. As these rates are often difficult to obtain, we estimated on-rates by assuming a diffusion limited process as described in section 2.3 and calculated off-rates from the equilibrium relationship

$$K_D = \frac{k_{off}}{k_{on}}. \tag{S1}$$

The rates of the model are listed in the main paper in Table 2.

2.2 Model analysis

Once the model was fully defined, we solved it with the *Multibind* approach as described in the main paper and obtained a set of thermodynamically consistent rates (Table 2 in the main paper and Table S5).

In order to obtain non-equilibrium steady-state transport estimates under a membrane potential, we modified the rates involving charges moving through the membrane as described in the main paper. Given the rates $k_{i,j}$, the master equations (4)

$$\frac{dp_i}{dt} = \sum_{j\neq i} k_{j,i}p_j - k_{i,j}p_i, \qquad 1 = \sum_i p_i. \tag{S2}$$

for the changes in state populations p_i were solved for the p_i under steady-state conditions, $dp_i/dt = 0$, using singular value decomposition (SVD). The net transition flux from state i to state j

$$J_{i,j} = k_{i,j}p_i - k_{j,i}p_j \tag{S3}$$

was calculated from the steady state populations p_i and p_j and the transition rates $k_{i,j}$ and $k_{j,i}$ between states i and j. $J_{i,j} > 0$ when there is net flux from i to j and $J_{i,j} < 0$ when the net flux is in the opposite direction; in equilibrium detailed balance $J_{i,j} = 0$ holds for all net fluxes.

For the equilibrium case, the thermodynamically consistent equilibrium binding constants were calculated from the consistent rates. They were close to the initial estimated parameters (Table S4), indicating that the chosen estimates were already close to a consistent model. The equilibrium model was solved for equal inside and outside sodium concentrations and for pH ranging from 0 to 15 in order to calculate the pH-dependence of the sodium binding free energy and the free energy difference between IF and OF conformation.

2.3 Diffusion limited ligand binding

For the antiporter model, order of magnitude estimates for ligand binding and unbinding rates are required. Given a free energy between the bound and unbound states, the ligand binding rate is used to calculate the unbinding rate or vise versa. With the diffusion coefficients of various ions known, we calculate the binding rate first. The binding process is treated as the diffusion of a ligand from bulk over a capturing surface. We assume that once the ligand crosses this surface binding occurs immediately. The radius of the capture surface, R, is set to reflect the patch size of the negatively charged surface near the binding pocket of an antiporter. The concentration of the ligand in equilibrium is treated as bulk concentration, c_0, far away from the capture

State 1	State 2	intrinsic rate constant and error ((M s)$^{-1}$)	reaction
OF(0)	OF(H^+)	19445000000.0 ± 358000000.0	OF(0) + H^+ $\longrightarrow$ OF(H^+)
OF(H^+)	OF(0)	6007.9 ± 4.1	OF(H^+) $\longrightarrow$ OF(0) + H^+
OF(0)	OF(Na^+)	3200439303.3 ± 3953.6	OF(0) + Na^+ $\longrightarrow$ OF(Na^+)
OF(Na^+)	OF(0)	167088412.3 ± 3259.9	OF(Na^+) $\longrightarrow$ OF(0) + Na^+
IF(0)	IF(H^+)	21589000000.0 ± 412000000.0	IF(0) + H^+ $\longrightarrow$ IF(H^+)
IF(H^+)	IF(0)	7888.7 ± 31.2	IF(H^+) $\longrightarrow$ IF(0) + H^+
IF(0)	IF(Na^+)	3199482412.5 ± 65046.1	IF(0) + Na^+ $\longrightarrow$ IF(Na^+)
IF(Na^+)	IF(0)	76739017.9 ± 2677.4	IF(Na^+) $\longrightarrow$ IF(0) + Na^+
IF(Na^+)	OF(Na^+)	4990.1 ± 18.5	IF(Na^+) $\longrightarrow$ OF(Na^+)
OF(Na^+)	IF(Na^+)	8006.2 ± 12.2	OF(Na^+) $\longrightarrow$ IF(Na^+)
IF(H^+)	OF(H^+)	8006.2 ± 12.2	IF(H^+) $\longrightarrow$ OF(H^+)
OF(H^+)	IF(H^+)	4990.1 ± 18.5	OF(H^+) $\longrightarrow$ IF(H^+)

Table S5: Thermodynamically consistent intrinsic rate constants, $k^{\circ}_{i,j}$, for the antiporter model, generated by the *multibind* rate projection procedure.

surface and zero at the capture surface. In cylindrical coordinates, the ligand concentration must satisfy Laplace's equation

$$\frac{1}{r}\frac{\partial}{\partial r}\left(r\frac{\partial c}{\partial r}\right)+\frac{1}{r^2}\frac{\partial^2}{\partial\theta^2}c+\frac{\partial^2 c}{\partial z^2}=0 \tag{S4}$$

subject to the following boundary conditions:

$$\begin{aligned} c &= 0; & z &= 0,\ r \le R \\ \frac{\partial c}{\partial z} &= 0; & z &= 0,\ r > R \\ c &= c_0; & z &= \infty,\ \forall r \\ c &= c_0; & r &= \infty,\ \forall z. \end{aligned}$$

The movement of the ligand follows down the concentration gradient and can be represented through the diffusion coefficient and the concentration from solving Equation S4. Integrating over the disc, the particle current through the capture surface is

$$\Phi=-\int_0^R r\,dr\int_0^{2\pi}d\theta\,D\left(\frac{\partial c}{\partial z}\right)_{z=0}, \tag{S5}$$

where

$$\left(\frac{\partial c}{\partial z}\right)_{z=0}=\frac{2c}{\pi}\frac{1}{\sqrt{R^2-r^2}}, \tag{S6}$$

as shown by Saito (5). The resulting particle current is then

$$\Phi = 4c_0RD, \tag{S7}$$

and the single ligand binding rate is estimated as

$$k = 4c_0RDN_A, \tag{S8}$$

where N_A is the Avogadro constant. The diffusion coefficient for free protons is taken as 9.3×10^{-5} cm^2/s (6) and 1.334×10^{-5} cm^2/s for sodium ions (7).

2.4 Cycle driving force of a simple antiporter transport cycle

The thermodynamic driving force of a cycle, χ, depends on the concentration gradients of the ions as well as the membrane potential $\Delta\Psi$,

$$\chi=\Delta\Psi\left(q_{H^+}-q_M\right)+kT\left(\ln\frac{[H^+]_{in}}{[H^+]_{out}}+\ln\frac{[M]_{out}}{[M]_{in}}\right). \tag{S9}$$

The first term depends on the charge difference between the transported species (proton H^+ and cation M) while the second term consists of the chemical potential difference, which depends on the concentrations of all species on both sides of the membrane. For electroneutral transport, the charge difference term is zero and the cycle driving force does not depend on the membrane potential difference. For electrogenic transport at fixed ion concentrations and pH, the driving force $\chi(\Delta\Psi)$ is a linear function of the membrane potential.

REFERENCES

1. Submeier, J. L., and C. N. Reilley, 1964. Nuclear Magnetic Resonance Studies of Protonation of Polyamine and Aminocarboxylate Compounds in Aqueous Solution. *Analytical Chemistry* 36:1698–1706.

2. Rohatgi, A., 2021. Webplotdigitizer: Version 4.5. https://automeris.io/WebPlotDigitizer.

3. Beckstein, O., and F. Naughton, 2022. General Principles of Secondary Active Transporter Function. *Biophysics Reviews* 3:011307.

4. Zwanzig, R. W., 2001. Nonequilibrium Statistical Mechanics. Oxford University Press, New York.

5. Saito, Y., 1968. A Theoretical Study on the Diffusion Current at the Stationary Electrodes of Circular and Narrow Band Types. *Review of Polarography* 15:177–187.

6. Agmon, N., 1995. The Grotthuss Mechanism. *Chemical Physics Letters* 244:456–462.

7. 1978. CRC Handbook of Chemistry and Physics (Online). *CRC handbook of chemistry and physics (Online)* .
